# Patients' Associations as Co‐Creators of Knowledge: Insights From a Community‐Based Participatory Research Study (ProSafe Project)

**DOI:** 10.1002/hsr2.72037

**Published:** 2026-03-18

**Authors:** Francesca Moretti, Silvia Colpo, Sara Montresor, Daniela Facchinello, Raffaella Robello, Mariangela Mazzi, Stefano Tardivo, Lisa Stagi, Luisa De Stefano, Andrea Zotti, Luigi Ambroso, Cristina Destro, Salvo Leone, Davide Petruzzelli, Ugo Moretti, Michela Rimondini

**Affiliations:** ^1^ Department of Neuroscience, Biomedicine and Movement University of Verona Verona Italy; ^2^ Department of Diagnostics and Public Health University of Verona Verona Italy; ^3^ Roche S.p.A., Monza (Monza e Brianza) Monza Italy; ^4^ Federazione delle Associazioni Emofilici ONLUS FEDEMO Rome Italy; ^5^ Women Against Lung Cancer in Europe ONLUS WALCE, Orbassano Turin Italy; ^6^ Associazione Nazionale per le Malattie Infiammatorie Croniche dell'Intestino (A.M.I.C.I. ETS) Milan Italy; ^7^ La Lampada di Aladino ETS Brugherio Monza e Brianza Italy

**Keywords:** co‐creation of knowledge, Community‐Based Participatory Research (CBPR), partnership, patient and public involvement and engagement (PPIE), patients' associations, survey co‐creation

## Abstract

**Background and Aims:**

Patient and public involvement and engagement (PPIE) in research is crucial for producing knowledge that is meaningful to the participating community. Sharing PPIE principles into practical tools and strategies enriches the development of participatory research methodologies. This article describes the co‐creation process of *ProSafe*, a Community‐Based Participatory Research (CBPR) project conducted in Italy, which explores community perspectives on the reorganization of territorial healthcare toward proximity care. The aims are: (1) to describe the co‐creation process, (2) to examine its successes and challenges, and (3) to propose strategies and practical recommendations for fostering partnerships and engagement throughout all research stages.

**Methods:**

The project is based on an explanatory sequential mixed‐method design. A Patient Safety Council (PSC) acted as a co‐researcher. The five‐level spectrum of public engagement developed by the International Association for Public Participation (IAP2) guided the analysis of the decision‐making power balance between academics and the PSC at each phase. Emphasis was given to the process of survey co‐creation. A SWOT (Strengths, Weaknesses, Opportunities, Threats) analysis was performed to identify strategies and practical recommendations to strengthen engagement.

**Results:**

The SWOT analysis results informed the development of OPERA‐PACT (Opening a Participatory and Equitable Research Agreement based on Partnership, Awareness, Collaboration, and Trust), a framework co‐created with the PSC to formalize shared principles, values, and attitudes that sustain collaborative partnerships. The framework also includes a commitment to continuous monitoring, verification, and feedback to reinforce partnership over time.

**Conclusions:**

All instruments, strategies, and methodological insights proposed in the article may serve as practical support tools to strengthen the voice of communities in research and to contribute to the production of valid and translational results. Implementing the OPERA‐PACT framework may further help align team members with PPIE principles and lay the foundation for effective and sustainable collaboration.

## 1 Introduction

Patient and public involvement and engagement (PPIE) in healthcare research have become increasingly recognized over the years as crucial to enhancing the relevance and validity of produced evidence [1]. Aligning research purposes and methods with the needs and perspectives of key stakeholders is essential to promote the practical application of results and to guide improvements that are genuinely valuable for the targeted community [2, 3]. Adopting participatory research encompasses various research designs [4]. One form of participatory research is community‐based participatory research (CBPR), defined as “an orientation to research often focused on health‐related issues that equitably involves all partners, including researchers and community members, in all phases of the research process, from study design to dissemination” [4]. The core values of CBPRs are well described in the literature. A fundamental principle of CBPRs is based on a trustful and equitable partnership between academic researchers and community members aimed at co‐learning and co‐creating knowledge to be translated into practical benefits for the targeted population [5, 6]. This collaboration is frequently formalized through the establishment of Community Advisory Boards (CABs), which consist of expert representatives from the community actively engaged as co‐researchers, ensuring consideration of the perspectives of the target population [7].

This study aims to describe the co‐creation research process entailed in a CBPR project named ProSafe. The project involves collaboration among academic researchers, an advisory board comprising representatives from patients' associations for various diseases (co‐researchers), and a pharmaceutical company (pharma company). The main goal is to explore the perspectives of the community of healthy citizens, patients dealing with various diseases, and caregivers on opportunities and potential new medication safety challenges arising from a territorial healthcare reorganization based on the promotion of proximity care, including the development of telehealth and digitalization of care. Indeed, in Italy, an important reform measure aimed at reorganizing and strengthening territorial healthcare was introduced with Mission Health 6 of the Italian National Recovery and Resilience Plan [8]. Crucial points of the reform are creating community‐based facilities such as Community Hospitals, further developing home care services, and expanding telehealth. These innovations, also promoted by other countries, aim to place patients at the heart of the healthcare system and ensure equitable, personalized, and effective access to prevention and disease management, specifically for chronic conditions [9]. Besides the potential benefits of these organizational solutions, there may be safety concerns, including emerging challenges in medication management. For example, ensuring patient safety in the home setting presents a significant challenge, particularly when comorbidities require complex pharmacological management [10]. Public perspective on opportunities and potential threats of the upcoming territorial reorganization is valuable to ensure truly patient‐centered care and proactively identify and implement actions to safeguard medication safety in this emerging proximity healthcare context. Moreover, public engagement at this level may lead to research findings capable of influencing choices at a political‐programmatic level, enabling a high level of community engagement in health decisions.

Despite the growing body of evidence on implementing participatory research and CBPR initiatives across various contexts, exchanging experiences regarding practical tools that support the application of fundamental principles in practice is valuable for providing insights into how PPIE in research can maximize its impact [11]. Additionally, insights into potential challenges and corresponding strategies to address them, as identified by community partners, are essential to ensure a genuinely participatory approach throughout all project phases [12, 13]. This is particularly relevant in the context of ProSafe. Engaging the community in examining how the transition toward proximity care and digitalized health services reshapes the roles of patients and caregivers in medication management and safety is crucial to capturing real‐world experiences, anticipating emerging safety issues, and co‐designing solutions that reflect the needs and expectations of those directly affected by this ongoing transformation in healthcare. Moreover, the ProSafe project addresses a healthcare aspect requiring the engagement of the entire community beyond the context and needs that characterize each disease. This approach, while allowing the integration of diverse perspectives that enrich the production of valuable data, also poses several challenges in maintaining a high level of community engagement. Insights on promoting and preserving active collaboration between researchers and community co‐researchers while appreciating the diverse cultures and approaches used to treat diseases in different patient associations may be a valuable perspective for further advancements in participatory research approaches. Accordingly, the primary aim of this article is threefold: (1) to describe the co‐creation process of the ProSafe project, with an emphasis on the actions and practical tools that supported the participatory approach and survey co‐creation process; (2) to explore the perspectives of community representatives from patient associations (Patient Safety Council, PSC) and of researchers on the successes and challenges of the partnership and engagement process experienced during the ProSafe study; and (3) to propose a synthesis of strategies and practical recommendations identified by the joint perspectives of community members and academics as essential for fostering partnerships at all research stages and promoting active collaboration between different patient associations.

## 2 Methods

### 2.1 The ProSafe Study Design

**Figure 1:**
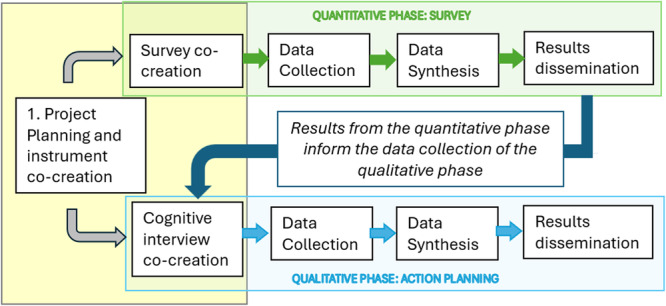
Main steps of the explanatory sequential mixed‐method design implemented in the ProSafe study.

The ProSafe study adopts a CBPR approach based on an explanatory sequential mixed‐method design. Results from a survey co‐created with the community (quantitative data) will inform the development of a set of cognitive interviews (qualitative phase), conducted with a sample of community members. Specifically, the semi‐structured interviews of the qualitative phase (data collection) will be built on the survey results to further explore the main emerging issues and to jointly identify—through co‐creation—the necessary actions to translate acquired insights into practical benefits. Therefore, the co‐creation process evolves from shared exploration (survey) to shared action planning (cognitive interviews), ensuring a synergistic integration between quantitative and qualitative data. Figure 1 provides a synthesis of the main steps of the project.

The data collection stage for the quantitative phase has concluded, and team members are working on data synthesis and results dissemination. The evolution of the action planning stage will be defined together with the PSC in the coming months. The study was conducted in accordance with the Declaration of Helsinki and was approved by the Ethics Committee of the University of Verona.

### 2.2 The ProSafe Study Team

The ProSafe study arises from a research partnership between the University of Verona and the PSC, an advisory board made including presidents and board members of four national patient associations representing individuals affected by diseases with a significant impact on healthcare system provision and a notable burden on quality of life [14]. These include: chronic inflammatory bowel disease (AMICI), congenital coagulation disorders (FedEmo‐ Federation of Hemophilia Associations), and cancer (WALCE—Women Against Lung Cancer in Europe and “La Lampada di Aladino”).

**Figure 2:**
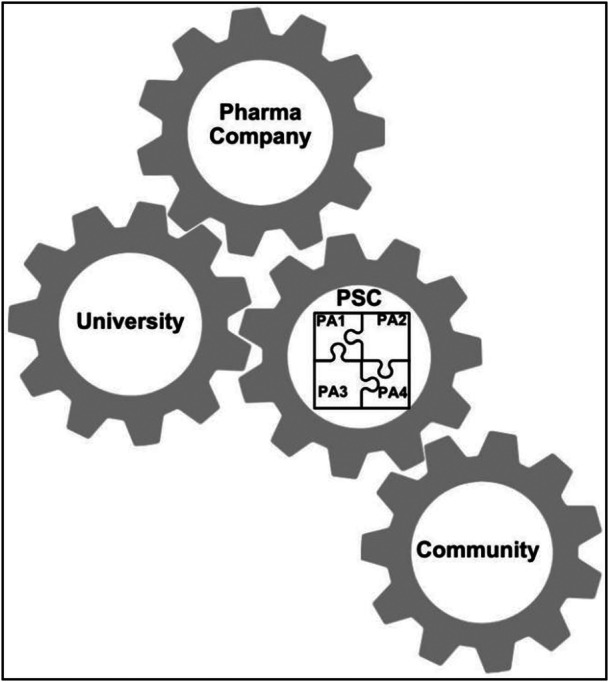
The complex dynamic network of relationships among ProSafe Team members.

The PSC was established in 2020 with the support of the pharmaceutical company Roche S.p.a. sharing the aim of raising awareness on patient safety and pharmacovigilance in pharmacological treatment [14]. The PSC plays an active role in facilitating the involvement of the communities it represents. Within the ProSafe study, the university team included experts in drug safety, proximity healthcare models, participatory research, and non‐technical skills such as communication, teamwork, and leadership. Roche S.p.a. facilitated the initial connection between academic partners and the PSC, supported contractual agreements with both parties according to their respective areas of expertise and promoted project coordination in line with the shared objectives. The pharma company acted as a neutral external facilitator throughout the process, providing logistical and organizational support and promoting balanced collaboration between the parties without being directly involved in research design, data collection, or decision‐making activities. Therefore, partnerships in the ProSafe study were established at multiple levels: between academics and PSC, between PSC and the represented communities, between the pharmaceutical partner and both academics and PSC, and between each patient association of the PSC (Figure 2). Establishing partnerships involves ensuring and fostering proficient and trusting collaboration within this complex, dynamic network of relationships to facilitate the optimal integration of all perspectives for the co‐creation of knowledge.

### 2.3 Co‐Creation Process: Levels of Engagement and Involvement Techniques at Each Decisional Point

**Figure 3:**
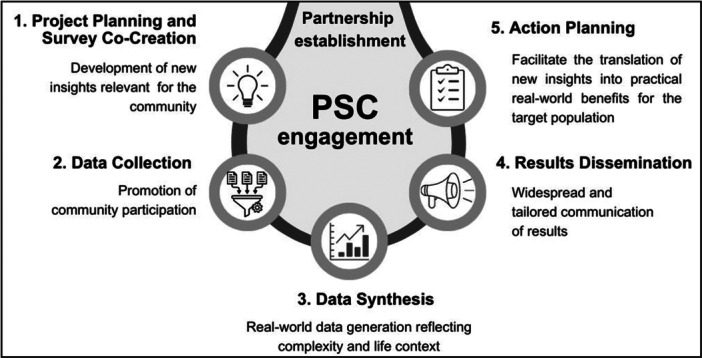
ProSafe stages and critical contributions of PSCs as co‐researchers.

As a co‐researcher in a CBPR project, the PSC was expected to bring its expertise to all stages of the research process, contributing to crucial research decisions and bringing significance to the results (Figure 3) [15].

The co‐creation of meaningful insights required continuously balancing decision‐making power to achieve integration between the scientific rigor of academic researchers and the experiential knowledge of the community. This equilibrium was essential for translating insights into practical actions closely aligned with the real needs and priorities of those directly affected by the ongoing transformation of care. According to the International Association for Public Participation (IAP2), stakeholder engagement moves along a continuum comprising five levels—from “inform” to “empower”—which reflect increasing degrees of collaboration and shared decision‐making [16]. A summary of the main characteristics of each level, the corresponding interaction between the academic researchers and co‐researchers, and the distribution of decisional power, is provided in Table 1.

**Table 1:** The spectrum of public participation (adapted from the IAP2 International Federation 2018, available at https://cdn.ymaws.com/www.iap2.org/resource/resmgr/pillars/Spectrum_8.5×11_Print.pdf).

Level	Characteristics	Information flow	Interaction (discussion)	Decision power
Inform	Balanced and objective information is provided to the community	Academic researcher → co‐researchers	Absent	Academic Researcher
Consult	Input is obtained from the community on analysis, alternatives, and decisions.	Co‐researchers→ academic researcher	Absent	Academic Researcher
Involve	Academic researchers work directly with the community to ensure its perspective is correctly understood and considered.	Bidirectional	Present	Academic Researcher
Collaborate	The community is a partner in the research process and each aspect of the decisions.	Bidirectional	Present	Shared
Empower	Community leads research decision‐making	Bidirectional	Present	Co‐researchers

For each of the five stages of the ProSafe project, major decision points were identified and mapped against these engagement levels to ensure an equitable distribution of decision‐making power between the researchers and co‐researchers. The corresponding involvement techniques implemented at each stage are summarized in Table 2.

**Table 2:** PSC level of engagement and decisional power balance at each decisional point.

Stage	Decisions	Steps	PSC level of involvement	Involvement technique
Project planning	What's the issue? What is/are the main research question/s?	Aims definition	Empower	Initial brainstorming between Pharma Company and PSC. Group discussion with academics.
	Which methodology will be used to answer the research question?	Study design	Empower	Initial brainstorming between Pharma Company and PSC; Group discussion with academics.
	Which population needs to be reached?	Recruitment criteria	Collaborate	Opinion‐based Group discussion
	Which method will be used to enroll the targeted population?	Recruitment method	Involve	Factual Group Discussion
	Which instrument will be used? What kind of data will be collected? How will the instrument be chosen/developed?	Instrument development (co‐creation)	Empower	Actions plan including individual and work groups
Data collection	How will the instrument be administered?	Data collection strategy	Collaborate	Factual and opinion‐based Group discussion
Data synthesis	How will data be analyzed? Which new insights are obtained from the data?	Data analysis	Inform	Concept maps
	Which hypothesis can be generated from the results? What are the implications of the research findings?	Data interpretation	Empower	Workshop
	How can data be effectively summarized? Which representation is best suited and comprehensible for the targeted population?	Data summary	Empower	Workshop
Results dissemination	Who is the target audience of the research findings? Which are vital messages, and how can they be effectively conveyed?	Results dissemination	Empower	Factual and opinion‐based Group discussion
Action planning	Which actions can be implemented to address the issue? How can findings be translated into concrete improvements?	Improvements definition	Empower	Next step (ongoing discussion among team members)
	How can actions be effectively implemented?	Implementation strategy	Collaborate	Next step (ongoing discussion among team members)

### 2.4 Project Planning and Survey Co‐Creation

**Figure 4:**
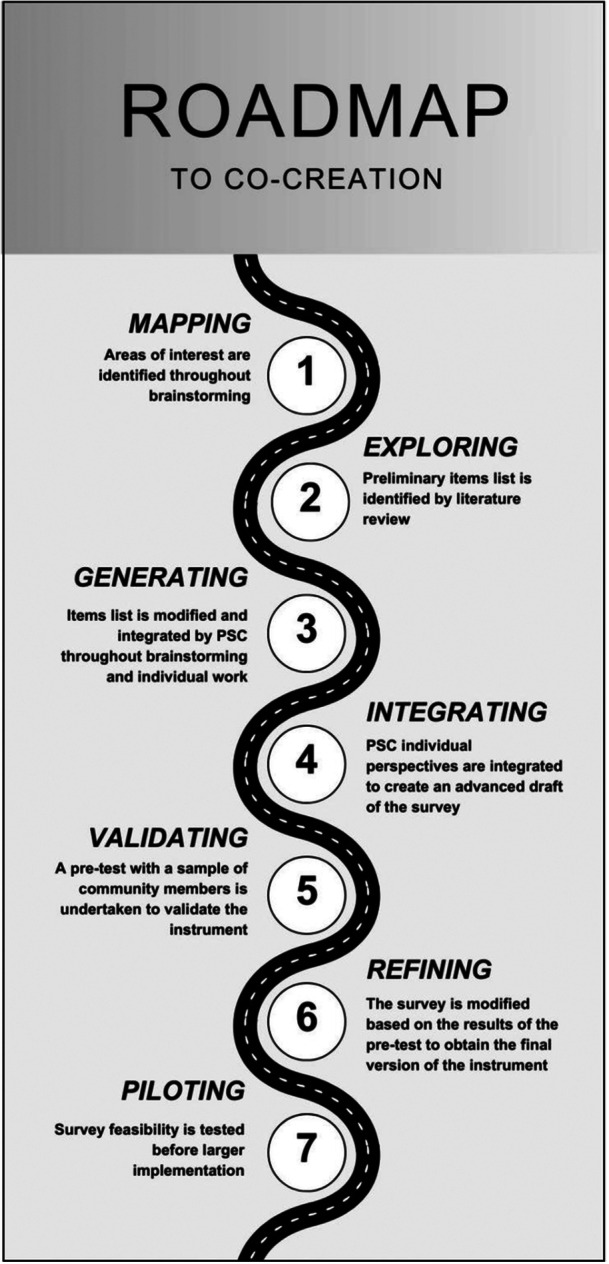
Survey co‐creation steps.

The process for survey co‐creation was developed based on a literature review and expert consensus (i.e., PSC and academics). The EU “Guidelines for the Development and Criteria for the Adoption of Health Survey Instruments” were used as a methodological reference to ensure validity and robustness [17]. The process followed seven main steps (Figure 4).

#### 2.4.1 Mapping

A brainstorming session was held to identify key areas of interest regarding the impact of proximity medicine on drug safety from the PSC perspective. Two main themes emerged: (i) the reorganization of the proximity network and the shift of drug administration from hospitals to community settings, and (ii) the digitalization of care and its implications for drug safety.

#### 2.4.2 Exploring

Academic members drafted an initial list of items based on the literature and national regulations relevant to proximity healthcare. Moreover, essential sociodemographic and other independent variables were included for data stratification.

#### 2.4.3 Generating

The draft survey was discussed with the PSC to refine items and ensure completeness. A participatory and open discussion allowed the PSC to suggest any modifications and additions. During this phase, members were encouraged to adopt a reflective and constructive attitude toward the proposed items, critically examining their clarity, relevance, and alignment with the project aims. After the meeting, each PSC member was requested to work on the draft list individually over the following 2 weeks, thereby minimizing potential group bias and allowing the PSC to fully integrate its perspectives.

#### 2.4.4 Integrating

All the observations and suggestions from the PSC were incorporated to create an advanced version of the survey, which was then reviewed collectively to reach consensus on remaining issues.

#### 2.4.5 Validation

Each patient association of the PSC coordinated a pretest with six community members to involve their perspective in the co‐creation process and evaluate clarity, comprehensibility, and acceptability of the instrument. Participants of different ages, genders, and educational levels completed the paper‐based survey and provided structured feedback to guide final revisions.

#### 2.4.6 Refining

A joint meeting with the PSC and academic researchers was held to share the pretest results and develop the final version of the survey. During the fine‐tuning phase, the PSC had final authority over content and item prioritization, whereas the academic team retained responsibility for methodological aspects and the management of independent variables to ensure scientific rigor.

#### 2.4.7 Piloting

After the first 50 responses were collected, preliminary analyses were performed to assess participation rates, completeness, and data quality.

### 2.5 SWOT‐Based Qualitative Analysis of the Co‐Creation and PPIE Process

Team members' perspectives on the successes and challenges they experienced while participating in the ProSafe project were collected. Specifically, individual semi‐structured interviews were conducted with all members of the PSC, academic researchers, and team members of the pharma company to gather their perceptions. A total of 10 interviews were conducted (four members of the PSC, four academics, and two members of the Pharma company).

Each interview lasted approximately 45 min and included a set of guiding questions developed based on the SWOT (Strengths, Weaknesses, Opportunities, Threats) framework to facilitate a structured critical reflection on the co‐creation process [18, 19]. Specifically, the questions encompassed a general query about the co‐construction process (e.g., “What are the main values of the ProSafe co‐construction project?”), along with a series of questions designed to facilitate a critical analysis of the project. The emphasis was particularly on evaluating aspects such as partnership and PPIE (i.e., “Which strengths have contributed to establishing the partnership between team members?”, “What are the main barriers/obstacles?”, “What strategies may be further implemented to foster the partnership?”, “How would you value the level of engagement at each stage of the research process?”, “What obstacles/barriers may arise toward a higher level of engagement?”, and “What strategies may be further implemented to foster the level of PSC engagement?”).

Perspectives from all team members were jointly organized within a SWOT analysis by three researchers familiar with the SWOT framework and experts in qualitative analysis (F.M., M.R., and M.M.).

The SWOT analysis is a well‐established evaluation and planning tool that provides a structured framework for identifying Strengths, Weaknesses, Opportunities, and Threats influencing an organization or a specific project [20]. Strengths and weaknesses refer to the current state of the project, while opportunities and threats pertain to potential future situations, generally created by external factors. This systematic assessment helps in understanding the current state of the planned project, pinpointing areas for improvement.

Participants' responses were iteratively coded, grouped, and reviewed into thematic categories corresponding to the four SWOT domains implemented as a deductive thematic framework. The process was conducted following established qualitative thematic analysis procedures to ensure methodological rigor [21]. Accordingly, two researchers (F.M. and M.M.) independently coded the data, reaching full agreement through iterative comparison; in cases of uncertainty, a third researcher (M.R.) was consulted to reach consensus. A strategic plan and practical recommendations to seize opportunities and mitigate threats by leveraging strengths and minimizing weaknesses were then developed based on SWOT results. Finally, all PSC members were invited to validate the SWOT analysis and to identify related strategies and recommendations through aiterative feedback process. Individual observations and suggestions were collected, discussed collectively, and integrated until a final consensus was reached.

## 3 Results

Table 3 shows the results from the SWOT analysis.

**Table 3:** SWOT analysis results.

**S** **T** **R** **E** **N** **G** **H** **T** **S**	Research topic cutting across various diseases (i.e., organizational level of health service provision and patient safety) Multidisciplinary expertise among academic team members, ensuring expertise also in the adoption and promotion of non‐technical skills. Involvement of different patient associations with diverse cultural approaches to disease and various impacts on the Healthcare System, each bringing their real‐world experiences of the pathology they represent. High level of involvement since the definition of aims and early stages of project planning using different engagement techniques. Power balance between researchers and co‐researchers supported also by the role of the Pharma Company as facilitator between academic researchers and PSC. Regular meetings throughout all stages of the project providing opportunities for efficient exchange of skills and experiences, as well as capacity building. A rigorous scientific approach applied to survey co‐creation (survey content not biased by academic researchers' perspective) An appropriate balance between community interests and each patient's interest, enabled by the role of the PSC as a mediator of community involvement. A strong sense of belonging among PAs toward the project which fosters motivation, awareness, and capability in proposing the survey to the community, facilitating its acceptance and cooperation. Allows for a comparison between the healthcare workers' perspective and that of the entire community on the same research topic	**W** **E** **A** **K** **N** **E** **S** **S** **E** **S**	A time‐consuming and highly demanding process that necessitates significant commitment, resources, and motivation. Sustainability challenge arising from such a demanding process. The development of high‐level competencies is necessary to sustain comprehensive and adequate engagement throughout the entire process. Striking a balance between the need to develop a survey that is easy to administer and the necessity to encompass all perspectives for an accurate analysis of the research topic can be challenging.
**O** **P** **P** **O** **R** **T** **U** **N** **I** **T** **I** **E** **S**	Provide evidence targeting the entire community beyond the specific context and needs that characterize each disease. Generation of high‐quality and valid data representative of stakeholders' perspectives, significant to real‐world context and relevant to the community. Facilitate the presentation of evidence in a simple, non‐technical way accessible to the whole community, enabling the translation of evidence into increased awareness as well as practical and effective interventions. Dissemination of results through various channels (e.g., scientific conferences, scientific journals, local initiatives), including high‐visibility channels with the potential for significant impact, such as press conferences facilitated by the pharmaceutical industry. Increase patient associations' opportunity to influence health‐related decisions by providing them with evidence‐based data that may be used to establish a dialogue with institutions. Establishing a network among patient associations with opportunities for sharing experiences, resources, best practices, and initiating a synergy of actions that could be beneficial in other contexts or future projects. Fostering a reciprocal exchange of knowledge and expertise within an ongoing co‐learning process.	**T** **H** **R** **E** **A** **T** **S**	Overload of patient associations with various projects may pose a challenge to the long‐term commitment necessary for a CBPR project. Cultural barriers toward projects promoted by pharmaceutical industry. Each stakeholder (i.e., university, pharma company, and different patient associations) may be inclined to prioritize their ‘own interests' rather than achieving a balance and integrating leadership based on the project's requirements. Hierarchical relationships between academic researchers and co‐researchers that can result in a “false and façade” engagement. Feelings of not being up to the task by co‐researchers Feelings of inadequacy and demotivation, especially for patient associations with fewer resources or competencies to deal with the role of co‐researchers. Potential loss of data richness and imbalanced contribution to the co‐production of knowledge due to lower participation of patient associations with less experience in participatory research and lower influence at a political‐decisional level, who may feel inhibited in expressing their perspectives. Surveys may be perceived as too mentally demanding for patients already overwhelmed by their disease. Surveys perceived as boring and repetitive by the community, leading to lower participation or inaccurate completion. The survey may be perceived as challenging by the community due to the limited general knowledge of the topic under investigation (the ongoing reorganization of proximity care posed potential difficulties in identifying any safety threats).

The interpretive synthesis of the SWOT analysis led to the identification of a set of strategies and recommendations aimed at maximizing opportunities and reducing weaknesses. These underscore the pivotal role of establishing and cultivating a research climate characterized by four main pillars: (i) a commitment to partnership for project co‐creation, (ii) awareness of individual value, (iii) mutual collaboration, and (iv) reciprocal trust and respect. A concise synthesis of the main emerging themes within each dimension—illustrating how these pillars foster partnership and PPIE—is presented in the following sections, while the complete list of discussion elements identified through the participatory analysis is provided as Supporting Information (Supporting Information S1: Table S1).

### 3.1 Commitment to Partnership for Project Co‐Creation

The dimension of Partnership highlights the importance of building and maintaining a collaborative environment grounded in shared values, open communication, and balanced decision‐making power. Team members emphasized the need to approach each meeting by fostering a climate of openness and mutual respect, where every perspective is explicitly valued, and no opinion is dismissed as right or wrong. Creating such a space for a transparent and open dialogue requires adopting communicative strategies aimed at suspending judgments and encouraging active listening to all viewpoints.

A key facilitating factor was the involvement of an external mediator—in this case, the pharmaceutical company—whose neutral role helped sustain a power balance between academic and community partners and supported the appreciation of complementary expertise. The already established trust between the PSC and the pharmaceutical company proved useful in facilitating collaboration with academic researchers and maintaining continuity across the project.

Finally, participants stressed the importance of establishing a shared framework of principles and values before initiating the collaboration. This was achieved through the adoption of a “pact for research,” which served as a symbolic and operational agreement aligning all partners on the ethical and relational foundations of their co‐creation effort.

### 3.2 Awareness of Individual Value

The dimension of Awareness refers to the process of developing a shared understanding of roles, responsibilities, and values within the co‐research group, and of recognizing the unique contribution of each partner to the project's goals. Participants highlighted the importance of strengthening community members' awareness of their role as co‐researchers, fostering a sense of legitimacy and responsibility in contributing to the production of valid results.

A central element was the promotion of self‐efficacy, supporting participants' confidence in their ability to actively engage in research tasks through clear communication of responsibilities, motivational support, and continuous monitoring of challenges. The group also stressed the need for a structured framework defining levels of engagement and decision‐making power, to balance the scientific rigor of the academic team with the practical expertise of community representatives.

Maintaining awareness requires continuous training and capacity building, with regular assessment of emerging needs to sustain participation over time. To further consolidate engagement, participants recommended the establishment of Research Community Boards and networks of patient associations, aimed at cultivating specific skills for participatory research and amplifying diverse patient voices across diseases.

To mitigate potential weaknesses or threats, it was considered essential to nurture collective awareness of the representative role held by patient associations—encouraging them to move beyond personal perspectives toward a broader, community‐oriented view. Sharing progress at each stage, leveraging digital and visual tools to promote engagement, and monitoring possible imbalances in motivation or participation were also seen as key to sustaining long‐term commitment. The presence of an external facilitator played a supportive role in addressing any emerging dissatisfaction and maintaining a balanced and reflective research climate.

### 3.3 Mutual Collaboration

The dimension of Collaboration encompasses the strategies adopted to build a cohesive, inclusive, and productive working environment throughout the co‐creation process. Participants emphasized the importance of identifying a project manager capable of integrating methodological rigor with relational and communicative competence—someone able to bridge academic and community perspectives and ensure the synergy of action required for a participatory approach.

To enhance cooperation, team members recommended selecting engagement methods suited to the goals of each project phase and the desired level of community involvement. Opportunities for informal exchanges, such as in‐person gatherings or virtual coffee breaks, were also seen as essential to recreating the sense of connection typical of traditional face‐to‐face collaboration. Ensuring that team members “felt at home” contributed to mutual understanding and motivation.

Effective collaboration was also associated with clear and adaptive communication strategies. Avoiding overly technical language, explaining scientific terms, and using analogies or visual supports (e.g., graphics, metaphors, symbols) were considered key to maintaining inclusiveness and comprehension among all participants. Creating feedback opportunities and sharing results after each stage of the project helped sustain engagement and self‐efficacy, reinforcing a collective sense of progress.

To minimize weaknesses and prevent disengagement, participants underlined the value of introducing ice‐breaking and welcome rituals to reduce hierarchy and encourage participation from all members. Cultivating humility and mutual respect between academic researchers and co‐researchers was described as essential to building confidence and preserving balance. Structured opportunities for dialogue—such as regular reflection meetings—enabled early detection of emerging issues and supported continuous improvement.

Finally, participants emphasized promoting a broader “culture of collaboration” across stakeholders, including patient associations, academic teams, and industry partners. Sharing positive examples of cross‐sector collaboration, as in the ProSafe project, was viewed as a way to legitimize participatory research within the pharmaceutical and scientific community, strengthen networks, and expand access to shared resources and expertise.

### 3.4 Reciprocal Trust and Respect

The dimension of Trust captures the interpersonal and organizational conditions that sustain openness, inclusion, and long‐term commitment within the co‐creation process. Building trust requires recognizing the diversity of the research team as a resource rather than a challenge. Participants emphasized the importance of embracing heterogeneity—valuing each member's expertise and background—to create an environment where everyone feels equally appreciated and empowered to contribute.

Trust also relied on the ability to manage conflicts constructively. Disagreements were viewed not as obstacles but as opportunities to enrich the discussion and co‐produce creative, “win–win” solutions. This approach encouraged dialogue and mutual understanding, allowing the team to integrate multiple perspectives and maintain a sense of collective ownership over decisions.

To prevent potential overload and preserve long‐term engagement, participants highlighted the need to agree on realistic levels of community involvement for each research phase, taking into account resource availability and workload balance. Transparency in setting expectations and mutual respect for limits were considered essential to maintaining both motivation and sustainability.

Finally, trust was consolidated through continuous communication and shared reflection on methodological decisions. Openly discussing challenges, clarifying rationale, and reaching consensus on research choices helped transform potential tensions into opportunities for learning and quality improvement. Establishing this climate of open dialogue laid the foundation for a relationship grounded in mutual confidence and respect for each partner's unique expertise.

### 3.5 The OPERA‐PACT Framework

The findings from the SWOT analysis and the subsequent interpretive synthesis emphasized that maintaining a constructive and inclusive research climate is essential to sustain meaningful co‐creation over time. Such a climate relies on the appreciation of diversity, mutual recognition, shared responsibility, and the alignment of values toward a shared intent for the co‐construction of knowledge among all team members. To preserve and promote such a climate, the research team identified the need to introduce a formalized moment of collective reflection and commitment allowing all members to explicitly agree upon a set of principles, values, and attitudes that characterize a collaborative partnership. This structured moment was conceived as an opportunity to reinforce shared understanding, strengthen cohesion, and ensure that all partners begin the project aligned on the same ethical and relational foundations.

Accordingly, a research agreement tool based on the four identified crucial pillars (i.e., Partnership, Awareness, Collaboration, and Trust) and related strategies and recommendations, named “OPERA‐PACT” (Opening a Participatory and Equitable Research Agreement based on Partnership, Awareness, Collaboration, and Trust) was developed. The framework was designed to translate the four key identified pillars into practical and actionable components that can guide participatory teams in establishing a common ground and sustaining engagement throughout all project phases. Accordingly, the OPERA‐PACT serves as a structured yet flexible framework to be shared with all team members at the beginning of each participatory project functioning as a “pact for research” that aligns everyone with the right atmosphere and creates a foundation for efficient collaboration and effective PPIE throughout all phases of the project. Drawing on the metaphor of an orchestra—where musicians tune their instruments before a performance, the tool symbolizes the importance of harmony, coordination, and mutual listening in participatory research. The parallelism between team members and musicians of an orchestra was chosen following the PSC's suggestion to use figurative language to convey meanings toward more intuitive and immediate communicative channels, different from solely cognitive and verbal ones.

**Figure 5:**
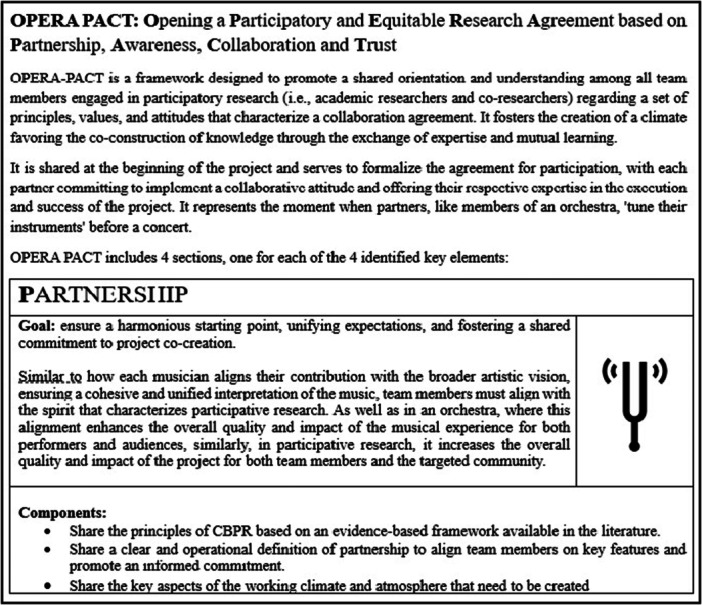
The first section of the OPERA‐PACT framework.

The tool is structured as a general framework that can be adapted by participatory research teams to their specific needs and contexts, while maintaining consistency with the overarching principles of community‐based participatory research (CBPR). The framework also encourages ongoing monitoring, verification, reflection, and feedback, to promote virtuous cycles of improvement and to foster long‐term involvement and partnership sustainability. Figure 5 illustrates the framework related to the first pillar (i.e., Partnership), while the complete version of the OPERA‐PACT is available as Supporting Information (Supporting Information S1: Table S2).

## 4 Discussion

A core value of the ProSafe research is the strong emphasis on project co‐creation with the community. This approach contrasts with traditional research methods, where knowledge is solely produced by academics (possibly through community consultations) and then adapted to be comprehensible and useful for the targeted population [22]. Specifically, the guiding principles of a CBPR approach include a strong focus on community‐defined problems, involvement at all stages of the research process, and maintaining partnerships throughout the employment of an interactive process [23].

In the ProSafe project, the PSC defined the primary research objectives, and the university was invited to collaborate with a specific mandate outlining the topic of the project to be developed. According to the literature, advisory boards have the potential to closely mirror the perspectives of the communities they represent, serving as an effective method for identifying valid priorities [24]. A systematic review conducted to explore which factors of participatory design are associated with additional positive outcomes beyond expected results recognized the "*impetus for the study coming from the organization*“ (rather than from researchers or both researchers and the university) as the most vital factor [25]. Although the importance of involving co‐researchers from the beginning of a project is acknowledged, the literature indicates that community engagement in defining goals is not very common; a recent comprehensive overview of the research partnership literature shows that approximately 423/787 (54%) of the included studies engaged stakeholders in identifying research questions [26]. Considering only the five reviews that implemented a CBPR approach, the percentage rises to approximately 60%, which is still far from the desirable high level of engagement outlined by CBPR principles.

The triangulated partnership involving the university, the network of patient associations (PSC), and the pharma company was viewed as a strength by all team members for fostering the partnership and ensuring a high level of engagement at all research stages. Expressly, the pharma company was acknowledged for facilitating the partnership between the University and the PSC, acting as a “reference point” for all patient associations. Simultaneously, the PSC played a crucial role in mediating community involvement. While community advisory boards (CABs) have been suggested as a means to establish a more balanced and efficient partnership between the university and the community, evidence shows that the creation and involvement of these bodies may be insufficient to ensure truly equitable partnership as persistent power dynamics and limited or merely formal participation can reduce their effectiveness [27, 28].

In this context, the role of the pharma company as a neutral external facilitator in aligning goals ensured that diverse expertise from both the university and the PSC was equitably valued and respected as essential within the research process, contributing to maintaining a balanced power distribution throughout the entire research process. Moreover, the process of efficiently identifying, approaching, and involving community members or their representatives in participatory research is complex and time‐consuming [29]. In the ProSafe study, the pharma company played a crucial role in facilitating this process by leveraging its established network of patient associations, such as the PSC, and mediating a trustworthy relationship between the university and the community.

A challenging aspect of the CBPR approach is engaging the community at all steps of the research project, ensuring that the cocreation process becomes a core value [15]. Despite the recognized importance of this crucial aspect, the literature shows that the real‐world implementation of this principle is still challenging. For example, CABs involved in a CBPR approach may often function as advisors (i.e., as a source of suggestions that may be accepted by academic researchers or not, retaining real decision‐making power) rather than as partners (i.e., co‐researchers with balanced decision‐making power) [7]. Moreover, in the previously mentioned umbrella review on research conducted in partnership with stakeholders, evidence from the included 18 reviews (corresponding to 870 individual studies) revealed that stakeholders' engagement varied according to the study stage. Specifically, mean percentages of engagement resulted in 30% for the stage of “disseminating research findings,” between 42% and 47% for the stages of “developing study design,” “data collection,” and “data analysis and/or interpretation,” and 54% in “identifying research question”; furthermore, in 85% of the review, authors evidenced a “lack of reporting on how and/or when stakeholders were engaged in the different phases of the research process” [26].

In the ProSafe study, reaching an agreement on the extent of PSC engagement at each research stage, ensuring its role as a valuable partner in the research process, emerged as a valuable strategy in the SWOT analysis, as was also emphasized by best practices to foster partnerships [26].

Measuring the level of involvement for each decision point according to a structured framework (e.g., the Spectrum of Public Participation of IAP2, which distinguishes the levels of inform, consult, involve, collaborate, and empower) helped to clearly define and control the distribution of decision‐making power throughout the project. Moreover, it may also foster project sustainability by allowing a flexible power distribution, reserving a higher level of involvement for decisions that play a significant role in ensuring validity. The PSC appreciated this efficient way of planning engagement as a means for ensuring continuous engagement while preventing patient associations overload, thereby preserving the maintenance of a trustful partnership. The valuable role of this strategy has also been confirmed in the literature [30]. Providing such detailed information may further help identify whether and how the potential benefits of participant involvement had a real impact on research effectiveness and outcomes [31, 32]. For example, high community engagement in problem definition and survey co‐creation likely contributed to the high percentage of people judging the survey as attractive in the pretest stage. At the same time, opinions regarding the survey length highlighted how a participatory approach may help ensure instrument feasibility. Moreover, the greater participation of the community subsample indicates how PSC made meaningful contributions to the recruitment stage.

Surveys are pivotal in gathering information from community members and creating valuable knowledge in health service organizations. However, the creation of surveys by researchers can introduce biases that limit the comprehensive exploration of community perspectives [33]. While surveys are efficient instruments for real‐world data exploration, their design should involve collaborative efforts involving researchers and the community [4]. This collaborative approach ensures that the survey instrument is culturally sensitive, relevant, and capable of capturing the nuances of community experiences. Despite the recognized importance of survey co‐creation, only a few studies have focused on this aspect [34]. The PSC in our project is actively involved in the co‐creation process, providing insights, perspectives, and feedback that enriches the survey instrument and enhance its validity in representing the community's viewpoint. However, evidence providing a detailed description of how community engagement is implemented is limited [35]. In our study, a step‐by‐step survey co‐creation process was used to enhance potential reproducibility. Moreover, the SWOT analysis allowed us to identify interesting elements for further enhancing the efficient implementation of surveys in a context where patient associations are overwhelmed by requests for survey participation, and patients may feel burdened by the impact of their disease, making it challenging for them to engage in mentally demanding activities such as survey completion. A proposed strategy was to creatively enhance dynamic interaction during survey compilation, decreasing monotony and increasing user‐friendliness. An additional strategy involved promoting community engagement in carrying out essential research tasks by organizing specific occasions with patient associations to connect with the community. These opportunities may include, for example, sharing interactive learning activities related to research involvement, balanced with informal recreational moments. The evidence supports the adoption of comparable strategies, as involving communities in creative activities has been demonstrated to contribute to the establishment of collaborative research partnerships [36, 37]. This approach shifts power dynamics toward the community, fostering their engagement and motivation.

The implementation of the SWOT analysis was a crucial opportunity, especially for reflecting on partnership and the project co‐creation process. Facilitating opportunities for critical discussions on the research process enabled the PSC to offer valuable feedback on both the strengths and weaknesses of research development. It served as a crucial, structured moment to reinforce the iterative nature of a CBPR approach and promote co‐learning [38]. Identified strategies and recommendations may help reinforce partnership identity, which is recognized in the literature as an essential step for ensuring the implementation of a participatory approach [39]. Accordingly, the results of the SWOT analysis may serve as a guiding document to enhance the participative approach during the subsequent stages of ProSafe to support the development of similar co‐created projects.

Despite the recognized value of fostering partnership and the development of several frameworks outlining key elements of the partnership process, to our knowledge, a guiding tool to commit all team members toward the establishment of a collaborative climate is still missing [40, 41]. The OPERA‐PACT tool was developed to fill this gap. The main aim of the framework is to provide a structured way to guide the process of sharing visions and building partnership identities so that all team members may be aligned and reinforce their synergic actions through a shared commitment to embracing attitudes and behaviors inspired by participatory research principles. It is structured to be adaptable in a flexible way to different participatory frameworks and different community populations. The tool is designed for utilization in the initial phase of participatory research, where the establishment of partnerships and equitable power distribution is crucial. Nevertheless, its focus on ensuring a feedback loop from communities and consistently analyzing research progress makes it a valuable resource for promoting an iterative approach and sustaining partnerships over time. Despite having been validated by ProSafe team members, external validation from both academic researchers and patient associations not involved in the current research is essential to further assess its usefulness and feasibility.

## 5 Conclusions

The ProSafe project not only contributes valuable insights into safety issues linked to therapeutic treatment but also may serve as an example of how to implement similar community‐engaged research. The emphasis on continuous community engagement, creative strategies for survey co‐creation, and the development of tools such as OPERA‐PACT may contribute to enhancing the development of high‐quality participatory research. As the project evolves, maintaining a strong partnership identity and addressing emerging challenges will be crucial for sustaining the impact and relevance of ProSafe in improving healthcare assistance and enhancing patient safety.

## Author Contributions

**Francesca Moretti:** conceptualization, data curation, formal analysis, methodology, investigation, project administration, resources, validation, visualization, writing – original draft, writing – review and editing. **Silvia Colpo:** conceptualization, methodology, data curation, formal analysis, writing – original draft, investigation, funding acquisition. **Sara Montresor:** conceptualization, investigation, methodology, formal analysis, data curation, writing – original draft. **Daniela Facchinello:** conceptualization, funding acquisition, investigation, methodology, project administration, resources, visualization, writing – review and editing. **Raffaella Robello:** conceptualization, methodology, investigation, funding acquisition, visualization, writing – review and editing, project administration, resources. **Mariangela Mazzi:** methodology, data curation, validation, formal analysis, supervision, visualization, writing – review and editing. **Stefano Tardivo:** investigation, writing – review and editing. **Lisa Stagi:** conceptualization, methodology, investigation, funding acquisition, visualization, resources, writing – review and editing. **Luisa De Stefano:** conceptualization, investigation, funding acquisition, methodology, visualization, writing – review and editing, resources. **Andrea Zotti:** conceptualization, investigation, funding acquisition, methodology, visualization, writing – review and editing, resources. **Luigi Ambroso:** conceptualization, investigation, methodology, writing – review and editing, visualization. **Cristina Destro:** conceptualization, investigation, methodology, visualization, writing – review and editing. **Salvo Leone:** conceptualization, investigation, methodology, visualization, writing – review and editing. **Davide Petruzzelli:** conceptualization, investigation, methodology, visualization, writing – review and editing. **Ugo Moretti:** conceptualization, methodology, data curation, investigation, validation, supervision, project administration, resources, writing – review and editing, software, visualization. **Michela Rimondini:** conceptualization, investigation, methodology, data curation, validation, supervision, formal analysis, visualization, resources, writing – review and editing. All authors have read and approved the final version of the manuscript. Prof. Francesca Moretti, the corresponding author, had full access to all of the data in this study and takes complete responsibility for the integrity of the data and the accuracy of the data analysis.

## Ethics Statement

The research involving human participants was reviewed and approved by the Institutional Review Board of the University of Verona. All procedures followed the ethical principles outlined in the 1964 Declaration of Helsinki and its subsequent amendments. The study was carried out in accordance with applicable guidelines and regulations.

## Consent

Informed consent was obtained from all subjects involved in the study.

## Transparency Statement

The lead author Francesca Moretti affirms that this manuscript is an honest, accurate, and transparent account of the study being reported; that no important aspects of the study have been omitted; and that any discrepancies from the study as planned (and, if relevant, registered) have been explained.

## Supporting information


**Supporting Table S1:** Proposed strategies for leveraging own strengths 1 and minimizing. **Supporting Table S2:** The OPERA‐7 PACT framework.

## Data Availability

The data that support the findings of this study are available from the corresponding author on reasonable request.

## References

[1] J. Brett , S. Staniszewska , C. Mockford , et al., “A Systematic Review of the Impact of Patient and Public Involvement on Service Users, Researchers and Communities,” Patient ‐ Patient‐Centered Outcomes Research 7, no. 4 (2014): 387–395. 25034612 10.1007/s40271-014-0065-0

[2] M. Cargo and S. L. Mercer , “The Value and Challenges of Participatory Research: Strengthening Its Practice,” Annual Review of Public Health 29, no. 1 (2008): 325–350. 10.1146/annurev.publhealth.29.091307.083824 18173388

[3] F. Jotterand , R. Spellecy , and R. Shaker , “The Rights (And Responsibilities) of the Public to Advance Health Through Research,” Archives of Public Health 79, no. 1 (2021): 198. 34784984 10.1186/s13690-021-00726-w PMC8594084

[4] L. M. Vaughn and F. Jacquez , “Participatory Research Methods—Choice Points in the Research Process,” Journal of Participatory Research Methods 1, no. 1 (2020), 10.35844/001c.13244.

[5] A. B. Israel , E. Eng , J. A. Schulz , and E. Parker . Methods for Community‐Based Participatory Research for Health, 2nd ed. (Jossey Bass, 2012), https://www.wiley.com/en-us/Methods+for+Community-Based+Participatory+Research+for+Health%2C+2nd+Edition-p-9781118021866.

[6] P. A. Holkup , T. Tripp‐Reimer , E. M. Salois , and C. Weinert , “Community‐Based Participatory Research,” Advances in Nursing Science 27, no. 3 (2004): 162–175. 15455579 10.1097/00012272-200407000-00002 PMC2774214

[7] D. S. Newman , O. J. Andrews , S. M. Gayenell , C. Jenkins , J. M. Cox , and C. D. Williamson “Community Advisory Boards in Community‐Based Participatory Research: A Synthesis of Best Processes.” *Preventing Chronic Disease* 8, no. 3 (2011): A70. PMC3103575 21477510

[8] Ministero dell'Economia e delle Finanze (MEF) . “Piano Nazionale di Ripresa e Resilienza (PNRR) – Missione 6: Salute.” Rome. 2021, https://www.italiadomani.gov.it/it/strumenti/documenti/archivio-documenti/piano-nazionale-di-ripresa-e-resilienza.html#:~:text=Il%20Piano%20Nazionale%20di%20Ripresa,economico%20e%20sociale%20della%20pandemia.

[9] T. Kendzerska , D. T. Zhu , A. S. Gershon , et al., “The Effects of the Health System Response to the COVID‐19 Pandemic on Chronic Disease Management: A Narrative Review,” Risk Management and Healthcare Policy 14 (2021): 575–584. 33623448 10.2147/RMHP.S293471 PMC7894869

[10] S. Strube‐Lahmann , U. Müller‐Werdan , J. Klingelhöfer‐Noe , R. Suhr , and N. A. Lahmann , “Patient Safety in Home Care: A Multicenter Cross‐Sectional Study About Medication Errors and Medication Management of Nurses,” Pharmacology Research & Perspectives 10, no. 3 (2022): e00953. 35506209 10.1002/prp2.953 PMC9066068

[11] A. Arumugam , L. R. Phillips , A. Moore , et al., “Patient and Public Involvement in Research: A Review of Practical Resources for Young Investigators,” BMC Rheumatology 7, no. 1 (2023): 2. 36895053 10.1186/s41927-023-00327-w PMC9996937

[12] R. A. Davis , H. B. Leavitt , and M. Chau , “A Review of Interventions to Increase WIC Enrollment and Participation,” Journal of Community Health 47, no. 6 (2022): 990–1000. 35962868 10.1007/s10900-022-01131-2 PMC9375084

[13] V. Donisi , A. Gajofatto , M. A. Mazzi , et al., “A Bio‐Psycho‐Social Co‐Created Intervention for Young Adults With Multiple Sclerosis (ESPRIMO): Rationale and Study Protocol for a Feasibility Study,” Frontiers in Psychology 12 (2021): 598726. 33708157 10.3389/fpsyg.2021.598726 PMC7940381

[14] P. Bandiera , M. Gianetta , S. Leone , A. Lupi , D. Petruzzelli , and S. Bianco , “Patient Associations as Key Players in Pharmacovigilance: Results of an Italian Survey From the Patient Safety Council,” Pharmadvances 3, no. 3 (2021): 568.

[15] T. M. Luger , A. B. Hamilton , and G. True , “Measuring Community‐Engaged Research Contexts, Processes, and Outcomes: A Mapping Review,” Milbank Quarterly 98, no. 2 (2020): 493–553. 32428339 10.1111/1468-0009.12458 PMC7296434

[16] International Association for Public Participation . IAP2. “Advancing the Practice of Public Participation.” 2018, https://www.iap2.org/page/pillars.

[17] Eurostat . “Guidelines for the Development and Criteria for the Adoption of Health Survey Instruments.” Luxembourg: Publications Office of the European Union, 2005, https://ec.europa.eu/eurostat/web/products-manuals-and-guidelines/-/ks-cc-05-003.

[18] D. Leigh , “SWOT Analysis.,” in Handbook of Improving Performance in the Workplace, ed. R. E. Pritchard , and N. J. Hoboken (Wiley, 2009), 115–140.

[19] M. Chiodini , “Participatory Evaluation: Methods and Tools.,” in Preventing Violent Radicalisation in Europe, ed. P. Meringolo (Springer International Publishing, 2020), 173–187, 10.1007/978-3-030-52048-9_9.

[20] E. Gürel , “Swot Analysis: A Theoretical Review,” Journal of International Social Research 10, no. 51 (2017): 994–1006.

[21] M. E. Kiger and L. Varpio , “Thematic Analysis of Qualitative Data: AMEE Guide No. 131,” Medical Teacher 42, no. 8 (2020): 846–854. 32356468 10.1080/0142159X.2020.1755030

[22] C. Grindell , E. Coates , L. Croot , and A. O'Cathain , “The Use of Co‐Production, Co‐Design and Co‐Creation to Mobilise Knowledge in the Management of Health Conditions: A Systematic Review,” BMC Health Services Research 22, no. 1 (2022): 877. 35799251 10.1186/s12913-022-08079-y PMC9264579

[23] S. E. Collins , S. L. Clifasefi , J. Stanton , et al., “Community‐Based Participatory Research (CBPR): Towards Equitable Involvement of Community in Psychology Research,” American Psychologist 73, no. 7 (2018): 884–898. 29355352 10.1037/amp0000167 PMC6054913

[24] T. Conway , T.‐C. Hu , and T. Harrington , “Setting Health Priorities: Community Boards Accurately Reflect the Preferences of the Community's Residents,” Journal of Community Health 22, no. 1 (1997): 57–68. 9120047 10.1023/a:1025198924501

[25] P. L. Bush , P. Pluye , C. Loignon , et al., “Organizational Participatory Research: A Systematic Mixed Studies Review Exposing Its Extra Benefits and the Key Factors Associated With Them,” Implementation Science 12, no. 1 (2017): 119. 29017557 10.1186/s13012-017-0648-y PMC5634842

[26] F. Hoekstra , K. J. Mrklas , M. Khan , et al., “A Review of Reviews on Principles, Strategies, Outcomes and Impacts of Research Partnerships Approaches: A First Step in Synthesising the Research Partnership Literature,” Health Research Policy and Systems 18, no. 1 (2020): 51. 32450919 10.1186/s12961-020-0544-9 PMC7249434

[27] S. Safo , C. Cunningham , A. Beckman , L. Haughton , and J. L. Starrels , “A Place at the Table:” a Qualitative Analysis of Community Board Members' Experiences With Academic HIV/AIDS Research,” BMC Medical Research Methodology 16, no. 1 (2016): 80. 27401678 10.1186/s12874-016-0181-8 PMC4940842

[28] G. Nelson , M. Jenkins , B. Knox , et al., “Engaging People With Lived Experience on Community Advisory Boards in Community‐Based Participatory Research: A Scoping Review,” International Journal for Equity in Health 24, no. 1 (2025): 209. 40682074 10.1186/s12939-025-02573-5 PMC12273312

[29] A. Giachello “Making Community Partnerships Work: A Toolkit.” 2007, https://aapcho.org/wp/wp-content/uploads/2012/02/Giachello-MakingCommunityPartnershipsWorkToolkit.pdf.

[30] M. Bird , C. Ouellette , C. Whitmore , et al., “Preparing for Patient Partnership: A Scoping Review of Patient Partner Engagement and Evaluation in Research,” Health Expectations 23, no. 3 (2020): 523–539. 32157777 10.1111/hex.13040 PMC7321722

[31] K. Staley , S. A. Buckland , H. Hayes , and M. Tarpey , “The Missing Links”: Understanding How Context and Mechanism Influence the Impact of Public Involvement in Research,” Health Expectations 17, no. 6 (2014): 755–764. 23107054 10.1111/hex.12017 PMC5060928

[32] K. Staley , “Is It Worth Doing?' Measuring the Impact of Patient and Public Involvement in Research,” Research Involvement and Engagement 1, no. 1 (2015): 6. 29062495 10.1186/s40900-015-0008-5 PMC5598089

[33] D. B. Resnik and C. E. Kennedy , “Balancing Scientific and Community Interests in Community‐Based Participatory Research,” Accountability in Research 17, no. 4 (2010): 198–210. 20597018 10.1080/08989621.2010.493095 PMC3074227

[34] M. S. Goodman , V. L. Sanders Thompson , and N. Ackermann “Creating a Survey of Community Engagement in Research,“ 2021, https://www.pcori.org/research-results/2016/creating-survey-community-engagement-research. 39312613

[35] L. P. Forsythe , L. E. Ellis , L. Edmundson , et al., “Patient and Stakeholder Engagement in the PCORI Pilot Projects: Description and Lessons Learned,” Journal of General Internal Medicine 31, no. 1 (2016): 13–21. 26160480 10.1007/s11606-015-3450-z PMC4700002

[36] A. Malpass , A. Breel , J. Stubbs , et al., “Create to Collaborate: Using Creative Activity and Participatory Performance in Online Workshops to Build Collaborative Research Relationships,” Research Involvement and Engagement 9, no. 1 (2023): 111. 38057911 10.1186/s40900-023-00512-8 PMC10701968

[37] K. Broomfield , C. Craig , S. Smith , G. Jones , S. Judge , and K. Sage , “Creativity in Public Involvement: Supporting Authentic Collaboration and Inclusive Research With Seldom Heard Voices,” Research Involvement and Engagement 7, no. 1 (2021): 17. 33731228 10.1186/s40900-021-00260-7 PMC7968302

[38] J. Powers , S. A. Cumbie , and C. Weinert , “Lessons Learned Through the Creative and Iterative Process of Community‐Based Participatory Research,” International Journal of Qualitative Methods 5, no. 2 (2006): 120–130.

[39] J. O. Andrews , S. D. Newman , O. Meadows , M. J. Cox , and S. Bunting , “Partnership Readiness for Community‐Based Participatory Research,” Health Education Research 27, no. 4 (2012): 555–571. 20837654 10.1093/her/cyq050 PMC3396876

[40] K.‐Y. Huang , S. C. Kwon , S. Cheng , et al., “Unpacking Partnership, Engagement, and Collaboration Research to Inform Implementation Strategies Development: Theoretical Frameworks and Emerging Methodologies,” Frontiers in Public Health 6 (2018): 190. 30050895 10.3389/fpubh.2018.00190 PMC6050404

[41] M. E. Nyström , J. Karltun , C. Keller , and B. Andersson Gäre , “Collaborative and Partnership Research for Improvement of Health and Social Services: Researcher's Experiences From 20 Projects,” Health Research Policy and Systems 16, no. 1 (2018): 46. 29843735 10.1186/s12961-018-0322-0 PMC5975592

