# Supplementary material for: Patients' Associations as Co‐Creators of Knowledge: Insights From a Community‐Based Participatory Research Study (ProSafe Project)
Source: Health Sci Rep. 2026 Mar 18;9(3):e72037. doi: 10.1002/hsr2.72037 (PMC13097388; doi:10.1002/hsr2.72037)
Supplement: Supplementary file 1 — Supporting Table S1: Proposed strategies for leveraging own strengths 1 and minimizing. Supporting Table S2: The OPERA‐7 PACT framework. [file HSR2-9-e72037-s001.pdf]

***Supplementary Information Table 1S.*** *Proposed strategies for leveraging own strengths and minimizing weaknesses*

| **MAXIMIZING OPPORTUNITIES by…** | | |
|---|---|---|
| **…leveraging own strengths.** | · Identify a project manager capable of integrating the need for implementing a scientifically rigorous methodology with relational and communicative skills, fundamental to ensuring a synergy of action between researchers and co-researchers. | COLLABORATION |
| | · Foster awareness on Community key role as co-researchers to support active participation of all team members and ensure valid results. | AWARENESS |
| | · Actively support the self-efficacy of co-researchers in their research participation (i.e., fostering their belief in their ability to successfully engage in and contribute to research activities) by sharing clear tasks, providing motivational support, and monitoring challenges. | AWARENESS |
| | · Recognize all necessary steps of the project based on tasks to be accomplished and decisions that need to be made to achieve final goals. Reach an agreement on roles, responsibilities, and the desirable level of decision-making power for academic researchers and co-researchers at different stages of the process, as well as the timing to accomplish the required tasks. To ensure a clear definition of responsibilities, it is preferable to implement a structured framework to identify different levels of engagement across the continuum and reach an agreement on what each level implies in terms of decision-making power (e.g., The Spectrum for Public Participation developed by the International Association for Public Participation for the ProSafe study). This is essential to foster the integration between the scientific rigor provided by the University and the practical real-world expertise and experiences guaranteed by community representatives. | AWARENESS |
| | · Choose various engagement methods to be employed in different project phases based on the goals and the desired level of community involvement to be achieved. | COLLABORATION |
| | · Create opportunities for in-person meetings and establish informal moments for socializing before research meetings. The main aim should be to make people feel 'at home.' For remote teams, organize virtual coffee breaks where team members can informally chat about non-work-related topics. This contributes to the recreation of the informal interactions that typically occur in a traditional in-person meeting. | COLLABORATION |
| | · Approach each meeting by creating a climate where the free expression of each perspective is facilitated. All opinions should be explicitly addressed as important. Team members must be aware that there are no right or wrong opinions, only precious contributions. Communicative strategies need to be implemented to create a climate of suspending judgments and foster active listening to all perspectives. | PARTNERSHIP |
| | · In the results dissemination phase, rely on various professionals to ensure the most effective communication strategy based on the chosen channel (scientific conferences, scientific journals, local initiatives, or press conferences). | COLLABORATION |
| | · Leverage an external facilitator, such as a pharma company for the ProSafe project, which may play a key role in mediating the relationship between academic researchers and co-researchers. This external facilitator supports the maintenance of a power-decisional balance, appreciates, and promotes the complementarity of expertise. In the ProSafe project, the already established trusting relationship between the PSC and the pharma company also facilitated the partnership with academic team members. | PARTNERSHIP |

| | | |
|---|---|---|
| | · Share the principles of CBPR and partnership to align team members on key features and promote an informed commitment before initiating the project. Establishing a "pact for research" to promote an agreement among all team members regarding a set of principles, values, and attitudes that characterize a collaboration partnership. | PARTNERSHIP |
| | Ensure timely identification of emerging training needs to support community engagement and implement on an ongoing basis required capacity-building actions | AWARENESS |
| **…minimizing weaknesses** | · Encourage the establishment of Research Community Boards, similar to the PSC, fostering the formation of a network of Patient Associations. These associations, recognizing their significance in generating valid results, should gradually cultivate specialized research engagement skills encompassing capacity building and self-efficacy. This approach increases the potential for creating inclusive participatory research projects that address the unique requirements of each disease. Although executing, supporting, and implementing such projects entails added complexity, they are crucial for amplifying the diverse voices within the intricate landscape of the real-world patient community. | AWARENESS |
| | · Foster an environment that encourages the sharing and provision of available resources to empower team members in overcoming any obstacles they may face. | COLLABORATION |

3

| **AVOID OR REDUCE THREATS by…** | | |
|---|---|---|
| **…make the most on strengths** | · Embrace the heterogeneity and the diversities of the research team as a strength, highlighting each contribution, thus fostering an environment in which everyone feels valuable. | TRUST |
| | · Promote awareness of PAs' role as representatives of their community, emphasizing the responsibility associated with this role, which requires overcoming personal viewpoints to embrace a broader and more comprehensive perspective. | AWARENESS |
| | · Strategies for overcoming conflicts and negotiating creative 'win-win' solutions when an agreement is necessary are fundamental. Eventual divergences need to be welcomed as opportunities to enrich perspectives, ensuring that all viewpoints can be shared. | TRUST |
| | · Consider the opportunity to introduce a welcome ritual or use ice-breaking strategies to help people introduce themselves in both formal and informal ways, aiming to minimize eventual hierarchical approaches. | COLLABORATION |
| | · Project managers and academic researchers need to cultivate and promote a climate of humility where the emphasis is on different expertise rather than power positions. Academic degrees should serve as a means to potentially highlight skills and enhance confidence in the scientific rigor and quality of the study. Addressing each other by name while maintaining a professional relationship can help create an appropriate atmosphere. | COLLABORATION |
| | · Ensure effective communication between researchers and co-researchers by limiting the use of technical terms. Explain any scientific concepts or words that need to be used. Be creative to further facilitate and convey more immediate comprehension by implementing figurative language, such as metaphors, similes, and analogies, as well as using images such as visual aids, graphics, or illustrations. | COLLABORATION |
| | · Exploit technological tools and forms of communication alternative to the verbal channel for actions promoting capacity building (e.g., using visual and interactive supports that capture interest). | AWARENESS |

| | | |
|---|---|---|
| **…overcoming weaknesses** | · Share achieved results after each stage to emphasize project progress and sustain motivation. | AWARENESS |
| | · Enhance, through regular feedback, the value of community involvement for each research phase to support motivation and self-efficacy. | COLLABORATION |
| | · Reach an agreement on the level of community engagement for each research phase, taking into account resource availability. Ensure that the agreed-upon engagement level does not result in an overload situation for community partners, which could jeopardize long-term commitment and the sustainability of the project. | TRUST |
| | · Create opportunities to discuss and collect inputs from co-researchers on the project's progress in order to highlight strengths, consolidate the sense of belonging to the project and enable the timely identification of any issues. A structured evaluation on an ongoing basis (e.g. using a SWOT analysis) allows for the enhancement of partnership, engagement, and motivation, thereby improving the sustainability of the project. | COLLABORATION |
| | · Actively explore potential imbalances in engagement among co-researchers to facilitate the timely recognition of issues or obstacles (e.g., low resources, loss of motivation, feelings of inadequacy, etc.). In this context, the external facilitator may play a key role by serving as a neutral point of reference for addressing any dissatisfaction or other negative feelings. | AWARENESS |
| | · Promote the exchange and refinement of the team's understanding and consensus on any methodological choices to sustain community motivation and transform methodological challenges into opportunities for higher quality Establishing open communication forms the basis for building a relationship grounded in mutual trust in each individual's expertise. | TRUST |
| | · Encourage community engagement by assisting PAs in balancing essential, demanding project activities with enjoyable elements. This may include organizing community meetings to introduce the project and the team or proposing interactive learning activities balanced with informal recreational moments. These meetings serve to create more pleasant opportunities to engage communities in necessary research tasks. For the ProSafe project, this could involve conducting pre-test interviews rather than survey compilation. | COLLABORATION |
| | · Facilitate survey completion by introducing elements that can enhance dynamic interaction during compilation, making it less monotonous and more user-friendly. For example, create a technological interface capable of displaying a visual overview of the whole survey, including sections identified by intuitive symbols, the importance of each section for project goals, the number of items per section, etc. The use of colors may further enhance comprehension and motivation. | COLLABORATION |
| | · Promote a "culture of collaboration" with all stakeholders, including Pharma companies, involved in the research projects, by fostering the dissemination of positive partnership experiences. This sharing can highlight how the pharmaceutical industry can also contribute to the development and value of participatory research. For example, by making their expertise available, promoting the creation of a network of patient associations (such as the PSC), providing practical support in all stages of development, and facilitating access to technological resources, etc." | COLLABORATION |

***Supplementary Information - Table 2S*** *The OPERA-PACT framework*

## **OPERA PACT: O**pening a **P**articipatory and **E**quitable **R**esearch **A**greement based on **P**artnership, **A**wareness, **C**ollaboration and **T**rust

OPERA-PACT is a framework designed to promote a shared orientation and understanding among all team members engaged in participatory research (i.e., academic researchers and co-researchers) regarding a set of principles, values, and attitudes that characterize a collaboration agreement. It fosters the creation of a climate favoring the co-construction of knowledge through the exchange of expertise and mutual learning.

It is shared at the beginning of the project and serves to formalize the agreement for participation, with each partner committing to implement a collaborative attitude and offering their respective expertise in the execution and success of the project. It represents the moment when partners, like members of an orchestra, 'tune their instruments' before a concert.

OPERA PACT includes 4 sections, one for each of the 4 identified key elements:

<table>
<tr><td colspan="2">PARTNERSHIP</td></tr>
<tr><td>Goal: ensure a harmonious starting point, unifying expectations, and fostering a shared commitment to project co-creation.<br><br>Just as each musician aligns their contribution with the broader artistic vision, ensuring a cohesive and unified interpretation of the music, team members must align with the spirit that characterizes participative research. As in an orchestra, where such alignment enhances the overall quality and impact of the musical performance for both musicians and audience, in participatory research it similarly enhances the quality and impact of the project for both team members and the target community.</td><td>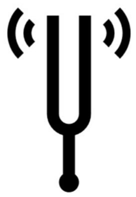</td></tr>
<tr><td colspan="2">Components:<br>• Share the principles of CBPR drawing on evidence-based frameworks described in the literature.<br>• Share a clear and operational definition of partnership to align team members on key features and promote an informed commitment.<br>• Share the key aspects of the working climate and atmosphere that need to be created</td></tr>
</table>

# AWARENESS

**Goal:** Empower each team member to recognize and appreciate their own valuable contribution, facilitating the establishment of expectations and alignment with individual roles and responsibilities.

As the orchestra conductor directs the entrance and harmonization of various instruments to create a melodious result, the scientific director must know how to appreciate the specific expertise of each team member and support the harmonization of real-world experience provided by the community with the scientific rigor ensured by academic team members in order to produce valid and scientifically sound evidence.

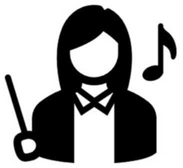

**Components:**

- Recognize and outline the expertise of each team member as a co-researcher, embracing heterogeneity and diversity as strengths, thus fostering an environment in which everyone feels valuable.
- Recognize all necessary steps of the project based on tasks to be accomplished and decisions that need to be made to achieve final goals. Reach an agreement on responsibilities and the desirable level of decision-making power for academic researchers and co-researchers at different stages of the process, as well as the timing to accomplish the required tasks. A structured framework can be implemented to clarify and reach an agreement on what each level of engagement implies in terms of decision-making power (e.g., The Spectrum for Public Participation developed by the International Association for Public Participation).
- Project managers commit to:
  i) Identifying emerging training needs to support on an ongoing basis community responsibility in research co-planning based on the defined level of involvement.
  ii) Implementing required capacity-building actions.
  iii) Encouraging co-researchers to engage in open communication in case of misalignment between responsibilities and competencies, in order to assertively and actively seek improvement actions.

# COLLABORATION

**Goal:** encourage all team members to support a participatory climate throughout the entire project

Just as an orchestra conductor shapes the dynamics, tone, rhythm, and balance among the various sections of the orchestra to bring harmony to the performance, the scientific director takes on the responsibility of sharing and defining an agreement on key attitudes and main actions needed to support a collaborative climate on an ongoing basis.

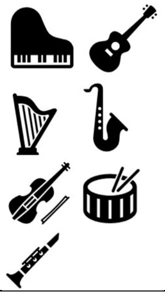

**Components:**

- Share best practices on key attitudes to be cultivated to foster a collaborative approach.
- Establish a commitment to regularly evaluate the established partnership, the level of community engagement, and the progress of the research in order to identify strengths and weaknesses and promote improvement strategies. A structured framework such as a SWOT analysis can be implemented to support the evaluation process.
- Commit to actively creating feedback loops, where stakeholders can provide ongoing input and updates. This iterative process can offer valuable support in reinforcing values and proactively identifying any challenges for timely and efficient management.

# TRUST

**Goal:** create and maintain an atmosphere of mutual trust and respect.

As in an orchestra, trust facilitates open communication and a shared understanding of musical interpretation, similarly, trust among team members eases the development of a shared vision for the project and ensures that everyone is moving in the same direction. A supportive and respectful environment enhances the group's ability to adapt seamlessly, allowing them to adjust their performance to respond to unexpected changes.

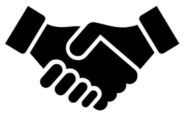

**Components:**

- Share key attitudes to cultivate a positive and respectful atmosphere. Feeling valued and respected supports motivation, fosters morale, and encourages team members to give their best effort, leading to a more engaging and high-quality performance.
- Commit to valuing any disagreement on specific research content as opportunities for enrichment, encouraging team members to always share their contributions, even when these differ significantly from others' perspectives
